# Genome Sequence of Salt-Tolerant *Bacillus safensis* Strain VK, Isolated from Saline Desert Area of Gujarat, India

**DOI:** 10.1128/genomeA.00671-13

**Published:** 2013-09-05

**Authors:** V. V. Kothari, R. K. Kothari, C. R. Kothari, V. D. Bhatt, N. M. Nathani, P. G. Koringa, C. G. Joshi, B. R. M. Vyas

**Affiliations:** Department of Biosciences, Saurashtra University, Rajkot, Gujarat, Indiaa; Department of Microbiology and Biotechnology, Christ College, Rajkot, Gujarat, Indiab; Department of Animal Biotechnology, Anand Agricultural University, Anand, Gujarat, Indiac

## Abstract

*Bacillus safensis* strain VK was isolated from the rhizosphere of a cumin plant growing in the saline desert of Radhanpar, Gujarat, India. Here, we provide the 3.68-Mb draft genome sequence of *B. safensis* VK, which might provide information about the salt tolerance and genes encoding enzymes for the strain’s plant growth-promoting potential.

## GENOME ANNOUNCEMENT

Deserts represent extreme environments for microorganisms, with conditions like high soil salinity, nutrient deficiency, high UV radiation levels, and strong winds. Desert soil microbial communities have shown unique and extraordinary diversity (1). *Bacillus safensis* VK, a Gram-positive, spore-forming, aerobic, chemoheterotrophic bacterium, was isolated from the rhizosphere of a cumin plant (*Cuminum cyminum*) from the saline desert of Radhanpar, Gujarat, India. It is observed to be a potent plant growth-promoting rhizobacterium (PGPR). *B. safensis* VK is a salt-tolerant microbe that is able to survive and grow in the presence of 14% NaCl and in the pH range 4 to 8.

The genomic DNA of *B. safensis* VK was isolated from a 24-h-old culture growing in nutrient broth using a commercial DNA isolation kit (GenElute bacterial genomic DNA kit, NA2110; Sigma-Aldrich). Whole-genome shotgun sequencing was performed using the 318-chip and 300-bp chemistry Ion Torrent PGM platform as per the manufacturer’s instructions. The draft genome of *B. safensis* VK showed the presence of 39 contigs of >200 bp in size when the sequence reads obtained were assembled into contigs using GS *de novo* Assembler v2.3.

The gene annotation and screening for RNAs were performed by submitting the sequences to the Rapid Annotations using Subsystems Technology (RAST) server (2). The circular chromosome of *B. safensis* VK comprises 3.68 Mb with 46.1% G+C content. We identified 3,928 protein-coding sequences (CDSs), of which 1,822 CDSs were assigned to one of the 457 RAST subsystems. The genome contains 73 tRNA genes.

The strain VK genome harbors the genes encoding enzyme 1-aminocyclopropane-1-carboxylate (ACC) deaminase that cleaves the precursor of plant hormone ethylene ACC into 2-oxobutanoate and NH_3_, resulting in the tolerance of the plant against stresses, such as salt (3), drafts (4), heavy metals, and polyaromatic hydrocarbons (PAHs) (5). *B. safensis* VK has the ability to produce enzymes of industrial importance, like amylase, protease, lipase, chitinase, and pectinase (6, 7). Information about various genes involved in the pathways related to the solubilization of phosphates, the production of indole 3-acetic acid, siderophore, hydrogen cyanide, and ammonia formation will give a better understanding of the PGPR properties of VK.

### Nucleotide sequence accession number.

The sequence of *B. safensis* VK has been deposited at GenBank under the accession no. AUPF00000000.
