# Genome Sequence of Salt-Tolerant *Bacillus safensis* Strain VK, Isolated from Saline Desert Area of Gujarat, India

**DOI:** 10.1128/genomeA.00337-14

**Published:** 2014-04-17

**Authors:** V. V. Kothari, R. K. Kothari, C. R. Kothari, V. D. Bhatt, N. M. Nathani, P. G. Koringa, C. G. Joshi, B. R. M. Vyas

**Affiliations:** Department of Biosciences, Saurashtra University, Rajkot, Gujarat, India; Department of Microbiology and Biotechnology, Christ College, Rajkot, Gujarat, India; Department of Animal Biotechnology, Anand Agricultural University, Anand, Gujarat, India

## ERRATUM

Volume 1, no. 5, e00671-13, 2013. Page 1, column 1, line 24: “…with 46.1% G+C content” should read “…with 41.6% G+C content.”

Page 1, column 1, line 27: “…contains 73 tRNA genes” should read “…contains 73 RNA genes.”

